# Estimations and projections of *Avena fatua* dynamics under multiple management scenarios in crop fields using simplified longitudinal monitoring

**DOI:** 10.1371/journal.pone.0245217

**Published:** 2021-01-15

**Authors:** Saeko Matsuhashi, Motoaki Asai, Keita Fukasawa

**Affiliations:** 1 CARC, National Agriculture and Food Research Organization, Tsukuba, Japan; 2 TARC, National Agriculture and Food Research Organization, Fukushima, Japan; 3 National Institute for Environmental Studies, Tsukuba, Japan; Feroze Gandhi Degree College, INDIA

## Abstract

Integrated weed management (IWM) is currently the most appropriate and effective method of agricultural weed control. To determine the most effective strategy, it is necessary to compare the effects of different control options and their rotation. *Avena fatua* (common wild oat) is one of the most common and economically threatening grass weed species of cereal crops worldwide. To examine the effects of non-chemical weed management options (farmland use, delayed sowing, and summer irrigation) on control of *A*. *fatua*, we recorded coverage levels and field conditions in 41 sites during the spring growing season of winter wheat for about 10 years. A transition matrix model was then constructed to project coverage levels of *A*. *fatua* under each management option using ordinal logistic regression. The results showed that farmland use had a remarkable effect on coverage; notably, planting of paddy rice and vegetables, which respectively eliminated the effect of coverage in the previous year and facilitated rapid convergence of coverage to 0%. Thus, although 90% of fields under continuous wheat cultivation were found to be at risk of *A*. *fatua* colonization, the risk was reduced to almost 0% with rotation of effective farmland use. As summer irrigation was also effective, more than 50% of wheat fields with the option continuously converged to no risk for *A*. *fatua* colonization. When the different management cycles were repeated, the effects were observed within 3 years, with a steady state reached in less than 10 years. Overall, these results suggest that simplified monitoring data could help decision-making on IWM, thereby helping to improve the efficiency of agricultural production.

## Introduction

Integrated weed management (IWM), defined as the combined use of multiple control tactics such as cultural, physical, biological, and chemical methods, is currently the most appropriate and effective method of agricultural weed control [1–3]. Since herbicide reduction contributes to reduce risks of environmental pollution and herbicide resistance [4–6] as well as to improve market value of products [7], crop protecting strategies alternative to chemical approach such as crop rotation and optimization of sowing time are important. To aid decision-making, and increase the efficiency of agricultural production and potential economic benefits for risk management, it is necessary to understand the effects of different weed management strategies and their rotation [8].

Previous studies have addressed the effects of various strategies and/or combinations of strategies on weed control. In general, field experiments allow data collection on weed abundance and/or biomass under different treatments, providing a direct understanding of the effects on weed control [6,9,10]. However, the number of treatments tends to be limited because of the time and labor required to carry out measurements. In contrast, categorical monitoring (on an ordinal scale of low, medium, and high) has recently been used to build projection models (density-structured models [11]) that allow direct analyses of the effects of different weed management strategies [12]. Obtaining simplified density data via field observations is much easier than measuring actual abundance, thereby allowing more data to be obtained from a greater number of sites over a longer period time, with the additional benefit of simultaneous analyses. Therefore, projection models based on simplified monitoring data have significant potential for developing weed management strategies and creating simulations of their long-term effects; however, few studies of such models have been conducted to date [12].

*Avena fatua* L. (common wild oat) is one of the most common and economically threatening weed species of cereal crops [13–15], and also one of the most herbicide-resistant weed species in the world [16]. To avoid further herbicide resistance, IWM is necessary for sustainable management of *A*. *fatua*. Several strategies have already been examined [17] such as crop rotation [18–21], changing sowing times [22,23], using competitive crops or cultivars [24,25], and increasing seeding densities [26], increasing soil water content to reduce the *A*. *fatua* seed bank [27,28], and combination of them in management cycles [18–20,29]. However, there is a limit to the number of experimental treatments that can be examined simultaneously or repetitively. Moreover, although data on the effects of each scenario and the required number of repetitions are required to optimize IWM strategies, determining the effects of numerous options and repetitions is difficult and requires simultaneous comparisons of each option across multiple years.

*A*. *fatua* has been regarded as a noxious weed of winter wheat and barley fields in Japan, especially the main island of Honshu, since the 1990s [30]. According to a questionnaire survey, *A*. *fatua* is recognized as noxious in 28 prefectures, with increasing weed damage in eight prefectures [31], however, there is currently no registered foliar-applied graminicide available for *A*. *fatua* [23]. Winter wheat fields are cultivated in rotation under “wheat-paddy rice,” “wheat-soybean-fallow-paddy rice,” or “wheat-vegetable” systems. In fields where the rotation sequence includes paddy rice, *A*. *fatua* populations tend to decrease because buried seeds are unable to survive in submerged paddy soil [28]. This rotation system is therefore regarded as a major and effective cultural control method of *A*. *fatua* in Japan; however, *A*. *fatua* continues to cause serious damage in upland winter-cereal systems.

In this study, we simultaneously evaluated the effects of multiple IWM strategies on the control of *A*. *fatua* by using accumulated coarse monitoring data of *A*. *fatua* coverage in a large number of crop fields in Japan. This study aimed to determine the following: 1) changes in *A*. *fatua* coverage in wheat fields without IWM strategies, 2) changes in coverage with different management strategies, and 3) changes in coverage over time with repetition of each management strategy. To determine the long-term effects, a transition matrix model was also established to describe the annual shift in *A*. *fatua* coverage from year to year. The results suggest that simplified monitoring data could be used to support IWM decision making and to increase the efficiency of *A*. *fatua* control.

## Materials and methods

### Study sites and data collection

**Fig 1:**
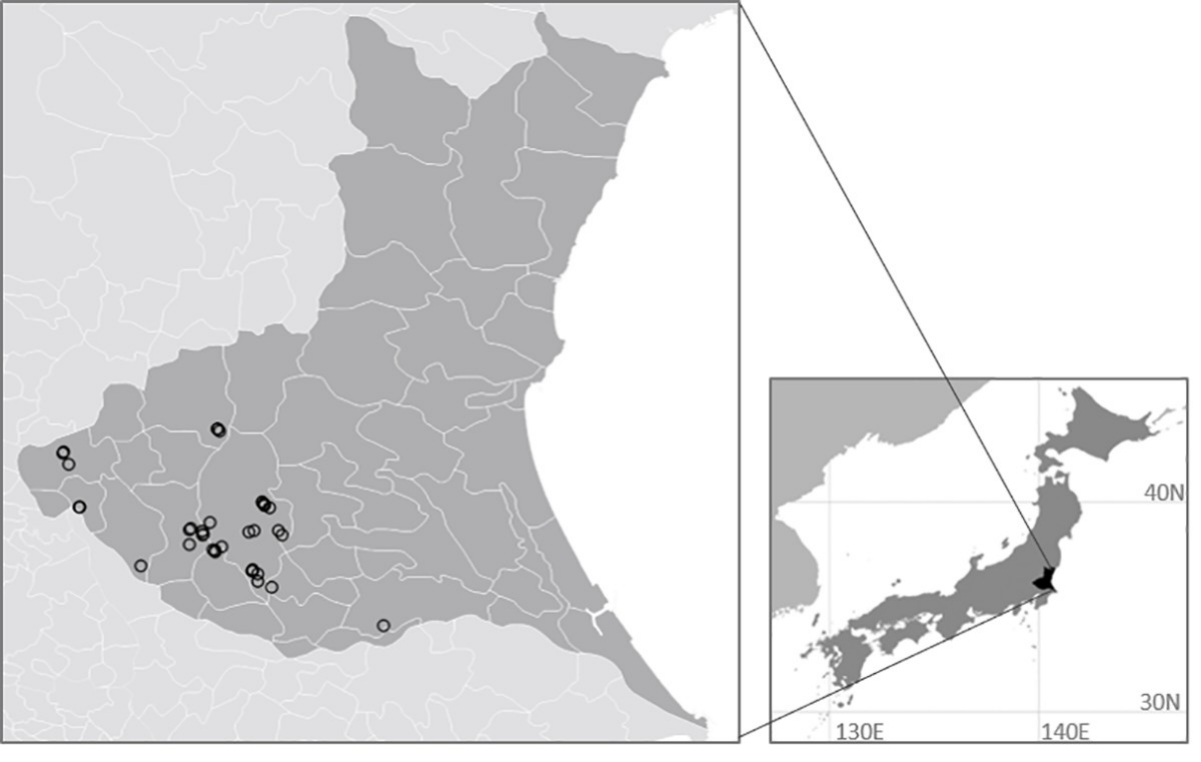
Locations of the 41 study sites in Ibaraki Prefecture, Japan. Points were converted into 1-km^2^ grid data.

In this study, field observations were carried out in a total of 41 fields in Ibaraki Prefecture, Japan from 1997 to 2009 (total of 425 visual observations; Fig 1, Table 1 and S1 Table). In Ibaraki Prefecture, many of the buried seeds were reported to germinate within a year [32]. In this area, wheat is usually sown in November and harvested in June, and delayed sowing from November to December has been considered to help eliminate the emerged *A*. *fatua* seedlings before wheat seeding, reducing the population density during the cropping season [23]. We selected wheat or barley fields where *A*. *fatua* colonized and conducted continuous annual visual observations. Surveys were conducted in May when *A*. *fatua* is in the flowering stage and easily observable, and is taller than wheat and barley. Coverage was classified into four levels as follows: 0 = “absent” (zero *A*. *fatua* found, degree of coverage 0%.), 1 = “low” (coverage less than 20%.), 2 = “medium” (coverage 20%–50%), and 3 = “high” (coverage more than 50%.). We also observed field and crop conditions and recorded the following. 1) farmland use comprised six categories: “wheat,” “barley,” “other crop (e.g. potato, vegetables, and, green manure crops),” “paddy rice,” “fallow with spring management (mainly tillage),” and “fallow without spring management.” Spring management was determined by investigations of soil conditions, weed size, and weed composition (S1 Table). Fields showing traces of tillage within a month, a small weed size and few winter weeds were classified as “fallow with spring management.” When no evidence of the above was found, the field was classified as “fallow without spring management.” 2) Delay in wheat sowing (binary) was determined by lower than expected growth. 3) Evidence of irrigation the previous summer (binary) was determined by observations of irrigation ditches, soil moisture, and weed composition.

**Table 1:** Geographical location (latitudes, longitudes, and address), observation year, and mean temperature (± standard deviation) of the 41 study sites during the winter wheat growing season (Dec–Apr).

Site ID	Lat (°N)	Lon (°E)	Years	Mean Tmp (Dec-Apr)	Address
1	36.23	140.04	1998–2009	7.19±0.67	Kuramochi, Akeno
2	36.23	140.04	1998–2009	7.19±0.67	Kuramochi, Akeno
3	36.23	140.04	1998–2009	7.44±0.68	Kuramochi, Akeno
4	36.23	140.04	1998–2009	7.19±0.67	Kuramochi, Akeno
5	36.23	140.04	2000–2009	7.33±0.69	Kuramochi, Akeno
6	36.19	139.78	1998–2009	6.91±0.61	Inamiya, Sowa
7	36.19	139.77	2005–2008	6.74±0.69	Inamiya, Sowa
8	36.17	139.78	1997–2008	6.89±0.59	Yagihashi, Sowa
9	36.11	140.11	1999–2009	6.90±0.66	Shibasaki, Tsukuba
10	36.11	140.11	1999–2006	6.68±0.60	Shibasaki, Tsukuba
11	36.10	140.12	1998–2009	6.89±0.63	Shibasaki, Tsukuba
12	36.10	140.11	1999–2008	6.78±0.63	Shibasaki, Tsukuba
13	36.10	140.12	1999–2009	6.87±0.66	Shibasaki, Tsukuba
14	36.10	139.80	1998–2009	7.01±0.61	Shimokohasi, Sakai
15	36.10	139.80	1998–2009	7.01±0.61	Shimokohasi, Sakai
16	36.10	139.80	1998–2009	7.01±0.61	Shimokohasi, Sakai
17	36.10	140.12	1998–2006	6.79±0.58	Konta, Tsukuba
18	36.07	140.02	1999–2009	6.57±0.65	Takasuka, Tsukuba
19	36.06	139.99	1997–2009	6.66±0.60	Nakatsuma, Mitsukaido
20	36.06	139.99	1998–2009	6.65±0.63	Nakatsuma, Mitsukaido
21	36.06	140.10	1998–2009	6.78±0.63	Teshirogi, Tsukuba
22	36.06	140.14	1998–2006	6.65±0.57	Umezono, Tsukuba
23	36.06	140.01	1998–2009	6.62±0.63	Nakakawasaki, Mitsukaido
24	36.06	140.01	1998–2009	6.68±0.63	Nakakawasaki, Mitsukaido
25	36.06	140.09	1998–2002	6.78±0.50	Teshirogi, Tsukuba
26	36.05	140.01	1998–2005	6.64±0.52	Kawasaki, Mitsukaido
27	36.05	140.15	1998–2009	6.77±0.63	Shimosasagi, Tsukuba
28	36.05	140.01	1998–2004	6.67±0.56	Higashi, Mitsukaido
29	36.04	139.99	1999–2009	6.73±0.65	Oyamato, Mitsukaido
30	36.03	140.04	1999–2003	6.57±0.62	Dai, Yawara
31	36.03	140.03	1999–2008	6.60±0.62	Fukuoka, Yawara
32	36.03	140.03	1999–2008	6.60±0.62	Fukuoka, Yawara
33	36.02	140.03	1999–2008	6.65±0.62	Fukuoka, Yawara
34	36.00	139.91	1997–2009	6.88±0.59	Yahagi, Iwai
35	35.99	140.10	1998–2007	6.67±0.63	Kukizaki, Tsukuba
36	35.99	140.10	1999–2008	6.58±0.62	Kukizaki, Tsukuba
37	35.99	140.10	1998–2008	6.60±0.60	Kukizaki, Tsukuba
38	35.99	140.10	1998–2007	6.75±0.63	Kamiiwasaki, Tsukuba
39	35.97	140.10	1998–2009	6.82±0.62	Shimoiwasaki, Tsukuba
40	35.96	140.13	1999–2003	6.76±0.61	Shironaka, Ushiku
41	35.90	140.32	1998–2009	6.67±0.63	Minamiota, Shintone

We also examined temperature, which is reported to affect the emergence of *A*. *fatua* [33,34]. To determine the effect of differences in temperature among fields and years, temperature data were obtained at a resolution of 1 km^2^ from Agro-Meteorological Grid Square Data, NARO (https://amu.rd.naro.go.jp/) [35,36]. Average temperatures during the growth period of *A*. *fatua* (December–April) were calculated for each field and observation year.

### Modeling

We used transition matrix models that describe the changes in weed states from one time step to the next [12,37] to project the coverage level of *A*. *fatua*. The coverage level of *A*. *fatua* in a given year depends on that in the previous year via the seed bank. A transition matrix model is applicable in such situations where a discrete state changes from year to year. In this study, the transition probability is assumed to depend on the management practices adopted and temperature. **x**_*ts*_ denotes a vector of management factors and temperature in year *t* at site *s*, and the transition matrix is expressed as follows:

$$P_{ts}=\left[\begin{array}{cccc} p_{00}(x_{ts}) & p_{01}(x_{ts}) & p_{02}(x_{ts}) & p_{03}(x_{ts})\\ p_{10}(x_{ts}) & p_{11}(x_{ts}) & p_{12}(x_{ts}) & p_{13}(x_{ts})\\ p_{20}(x_{ts}) & p_{21}(x_{ts}) & p_{22}(x_{ts}) & p_{23}(x_{ts})\\ p_{30}(x_{ts}) & p_{31}(x_{ts}) & p_{32}(x_{ts}) & p_{33}(x_{ts}) \end{array}\right]$$

Here, *p*_*ji*_ denotes the transition probability from level *i* to level *j*, and the subscripts 0, 1, 2, and 3 indicate respective levels of coverage. The sum of each column is 1. For the explanatory variables, **x**, we considered farmland use (6-level categorical variable), summer irrigation (binary variable), delayed sowing (binary variable), and temperature (continuous variable). The state probability vector for the four coverage levels, **q**_ts_, is given by the product of the transition matrix and the state probability vector for the coverage levels in the previous year, **q**_*ts*_ = **P**_*ts*_**q**_*t*-1,*s*_. The equilibrium state probabilities as *t* → +∞ are given by the right eigenvector associated with the dominant eigenvalue (*λ* = 1) of **P**_*ts*_.

To estimate the transition probability matrix and effects of the different weed management strategies on the transition probabilities of *A*. *fatua* in crop fields, we fitted mixed ordinal logistic models [38] to the observed coverage levels. These models are formulated as a set of threshold regression models on transition probabilities:

*p*_0*i*_(**x**_*ts*_) = f_0|1_(*i*, **x**_*ts*_),

*p*_1*i*_(**x**_*ts*_) = f_1|2_(*i*, **x**_*ts*_)—f_0|1_(*i*, **x**_*ts*_),

*p*_2*i*_(**x**_*ts*_) = f_2|3_(*i*, **x**_*ts*_)–f_1|2_(*i*, **x**_*ts*_),

*p*_3i_(**x**_*ts*_) = 1—f_2|3_(*i*, **x**_*ts*_),

where f_*j*|*j*+1_(*i*, **x**_*ts*_) is a function of the threshold probability between coverage levels *j* and *j*+1 depending on the previous year’s coverage level *i* and explanatory variables **x**_*ts*_. The threshold function is given by a mixed logistic regression model:

f_*j*|*j*+1_(*i*, **x**_*ts*_) = Logit(*β*_*j*|*j*+1_ –(*α*_*i*_ + **βx**_*ts*_ + *ε*_*s*_)).

Here, *β*_*j*|*j*+1_ denotes threshold coefficients of coverage levels *j* and *j*+1, which increases in the order *β*_0|1_ < *β*_1|2_ < *β*_2|3_, and *α*_*i*_ denotes ordered factors determining the dependence on the previous year’s coverage level *i*, with baseline *i* = 0 (*α*_0_ = 0 < *α*_1_ < *α*_2_ < *α*_3_ or *α*_3_ < *α*_2_ < *α*_1_ < 0 = *α*_0_). **x**_*ts*_ and **β** are vectors of explanatory variables and the corresponding regression coefficients, respectively, and ε_s_ is a site-level random effect with Gaussian prior ε_s_ ~ Normal (0, *σ*^2^).

The observed coverage level, *Y*_*ts*_ = *j*, conditional on the state of the previous year, *Y*_*t*-1,*s*_ = *i*, follows a multinomial distribution with probability vector **p**_.*i*_ = (*p*_0*i*_(**x**_*ts*_), *p*_1*i*_(**x**_*ts*_), *p*_2*i*_(**x**_*ts*_), *p*_3*i*_(**x**_*ts*_)). Model fitting was performed using the marginal maximum likelihood method with the *clmm2* function of the *ordinal* package [39] in R software version 3.5.1 [40]. The best model was selected using downward model selection with the Akaike information criterion (AIC) [41] and was used for the projection.

### Simulation of the effects of management strategies

To compare the effects of each management strategy and its repetition, we simulated temporal changes in the state probabilities of *A*. *fatua* coverage levels under different management scenarios using the best model. The management scenarios were as follows: repeated wheat cultivation, rotation of wheat and five other farmland uses, wheat with summer irrigation, and rotation of wheat with and wheat without summer irrigation. The initial level of *A*. *fatua* coverage was set at 0 or 3. The temporal changes across 20 years were then simulated. In all simulations, the random effect was fixed at zero to exclude the effects of differences among sites and to allow the average effects of each site to be determined.

We also calculated equilibrium state oscillation under different cycle options. When two different farmland uses are rotated from year to year, the probabilities of coverage levels oscillate between two states at equilibrium [42]. Given the transition matrices corresponding to two farmland use, **P**_1_ and **P**_2_, the probabilities of equilibrium coverage levels are given by eigenvectors of the two matrix products (**P**_2_**P**_1_) and (**P**_1_**P**_2_), respectively.

### Ethics statement

All field works (visual observations) were conducted on public roads and no permission was required under law in Japan. All information of site locations is coarse enough to protect landowners' privacy.

## Results

The difference in AIC value between the full model and the models that excluded the effects of delayed sowing and/or temperature was less than 2 (Table 2); thus, the models used in the subsequent projection analyses excluded these two variables. Although their effects were smaller than those of the remaining variables, delayed sowing and temperature had a negative and positive effect on coverage of *A*. *fatua*, respectively.

**Table 2:** Coefficients and AIC values of the mixed ordinal logistic regression models showing the effects of coverage in the previous year, summer irrigation, delayed sowing, farmland use, and temperature on coverage levels of *Avena fatua*.

Coverage in the previous year			Summer irrigation	Delayed sowing	Farmland use					Temp.	Threshold			AIC
Lv1	Lv2	Lv3			Barley	Other crop	Paddy rice	Fallow (management)	Fallow		Lv0|Lv1	Lv1|Lv2	Lv2|Lv3	
4.23	8.43	12.44	-1.39		0.22	-6.77	-18.79	-5.34	-1.60	0.31	-1.01	2.34	4.37	633.02
4.27	8.46	12.37	-1.44	-0.91	0.18	-6.86	-19.75	-5.43	-1.68	0.30	-1.19	2.17	4.23	633.12
4.26	8.47	12.46	-1.45	-0.98	0.21	-6.83	-19.75	-5.46	-1.63		-3.16	0.16	2.20	633.86
4.22	8.44	12.57	-1.39		0.26	-6.73	-18.79	-5.36	-1.54		-3.08	0.22	2.23	634.08
4.38	8.79	13.05		-0.85	0.26	-6.79	-19.78	-5.37	-1.67	0.32	-0.88	2.44	4.49	637.75
3.64	8.44	16.78	-0.90	-0.27						0.30	0.32	2.69	4.29	803.55
			-3.23	-0.39	-0.36	-6.40	-19.26	-4.28	-0.72	0.36	-0.42	2.05	3.45	914.20

**Fig 2:**
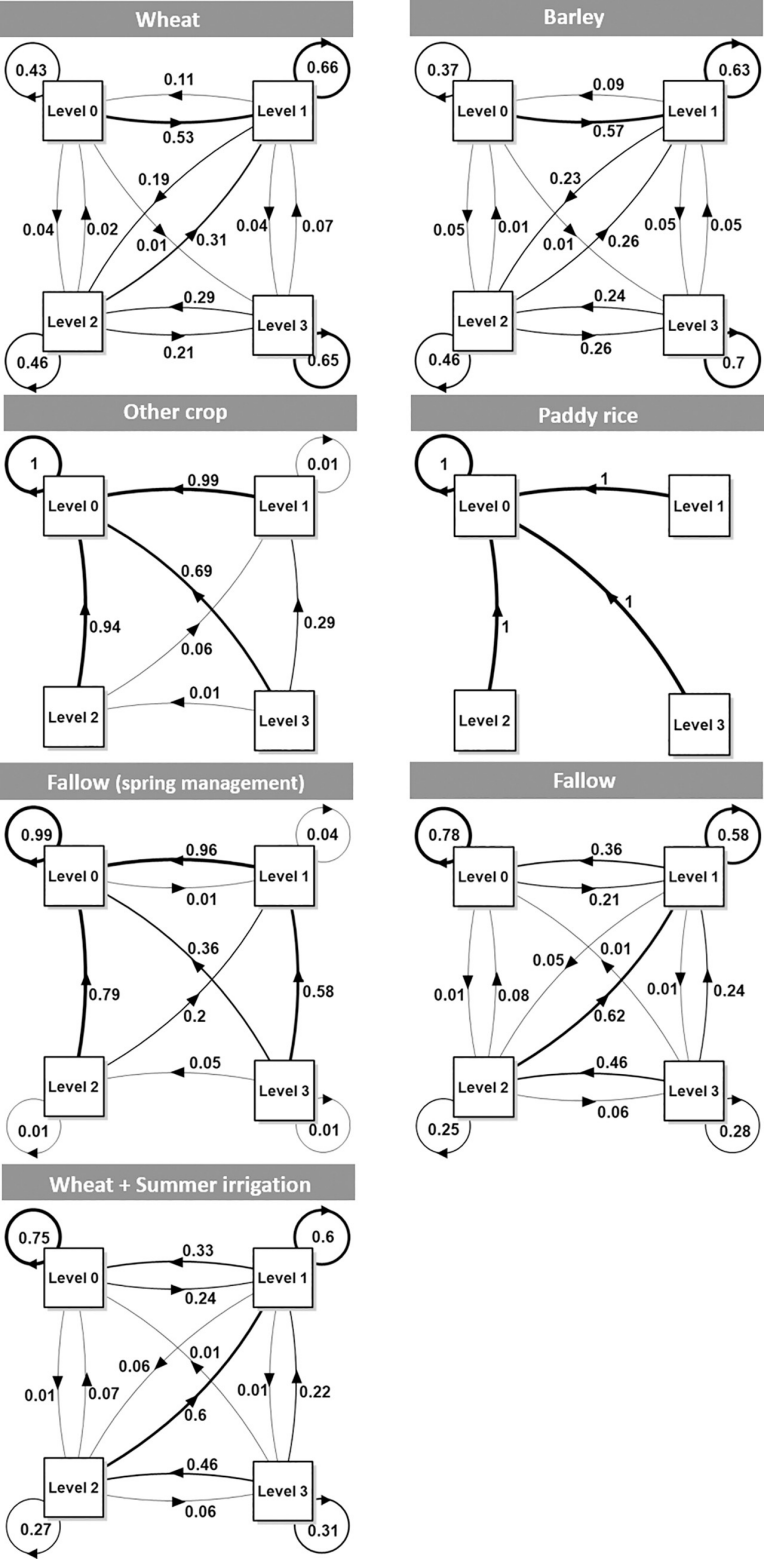
Transition probabilities of each level of *Avena fatua* coverage under six types of farmland use (wheat, barley, other crop, paddy rice, fallow with spring management, and fallow without spring management) and in wheat fields with summer irrigation. Levels 0, 1, 2, and 3 indicate zero, low, medium, and high coverage of *A*. *fatua*, respectively.

Farmland use had a remarkable effect on the coverage level of *A*. *fatua*, and under repeated “wheat” cultivation (Fig 2), about half of the state of level 0 shifted to level 1. In most cases, the increase or decrease was gradual (e.g. from level 0 to 1 or level 3 to 2) and the probabilities of skipping a level (e.g. from level 0 to 2 or level 3 to 0) were less than 0.1. Similar results were also observed in the “barley” fields (Fig 2). In contrast, when “other crops” were selected, level 0 was maintained, and sites with level 1 or 2 tended to decrease to level 0 with probabilities of 0.99 and 0.94, respectively, whereas those at level 3 tended to shift to level 0 or 1. When “paddy rice” which had more effect than the other options (Table 2) was selected, a consistent shift to level 0 was observed for level 1 to 3. Meanwhile, the effect of “fallow with spring management” was stronger than that of “fallow without spring management,” and under “fallow with spring management,” level 0 tended to be maintained, with a decrease in other levels. When “fallow without spring management” was selected, levels tended to decrease, although an increase was observed in some cases.

All other management options were also projected to affect *A*. *fatua* coverage. In wheat fields where summer irrigation was conducted, levels tended to decrease, with a probabilities of maintaining levels 0 and 1 of 0.75 and 0.6, respectively, and of maintaining levels 2 and 3 of 0.27 and 0.31, respectively. The probability of a shift from level 1 to 0 (0.3) was higher than that of a shift from level 0 to 1 (0.2), and although rare, a shift from level 3 to 0 was also projected (0.01).

**Fig 3:**
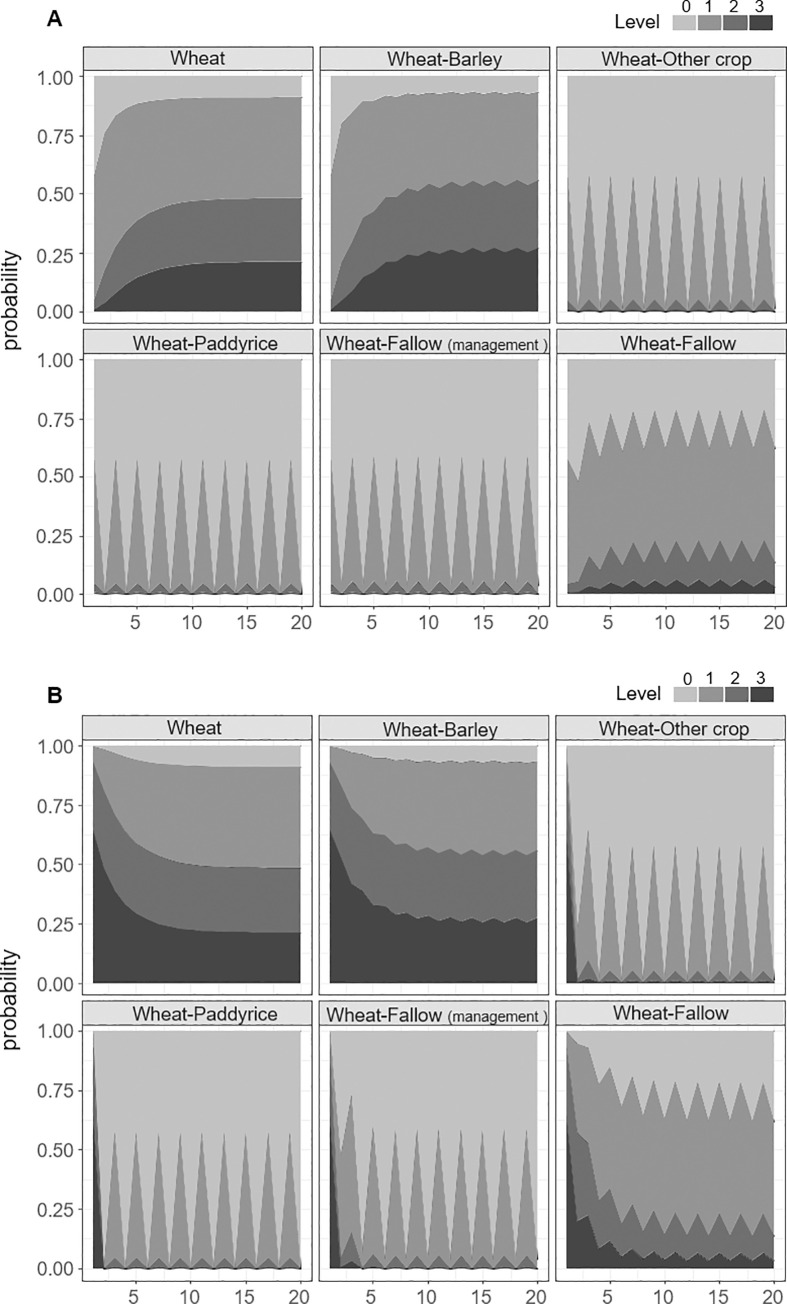
Projected state probabilities following repeated cycles of “wheat,” “wheat-barley,” “wheat-other crop,” “wheat-paddy rice,” “wheat-fallow with spring management,” and “wheat-fallow without spring management”. A) Change from level 0 (*A*. *fatua* is absent). B) Change from level 3 (high *A*. *fatua* coverage).

**Fig 4:**
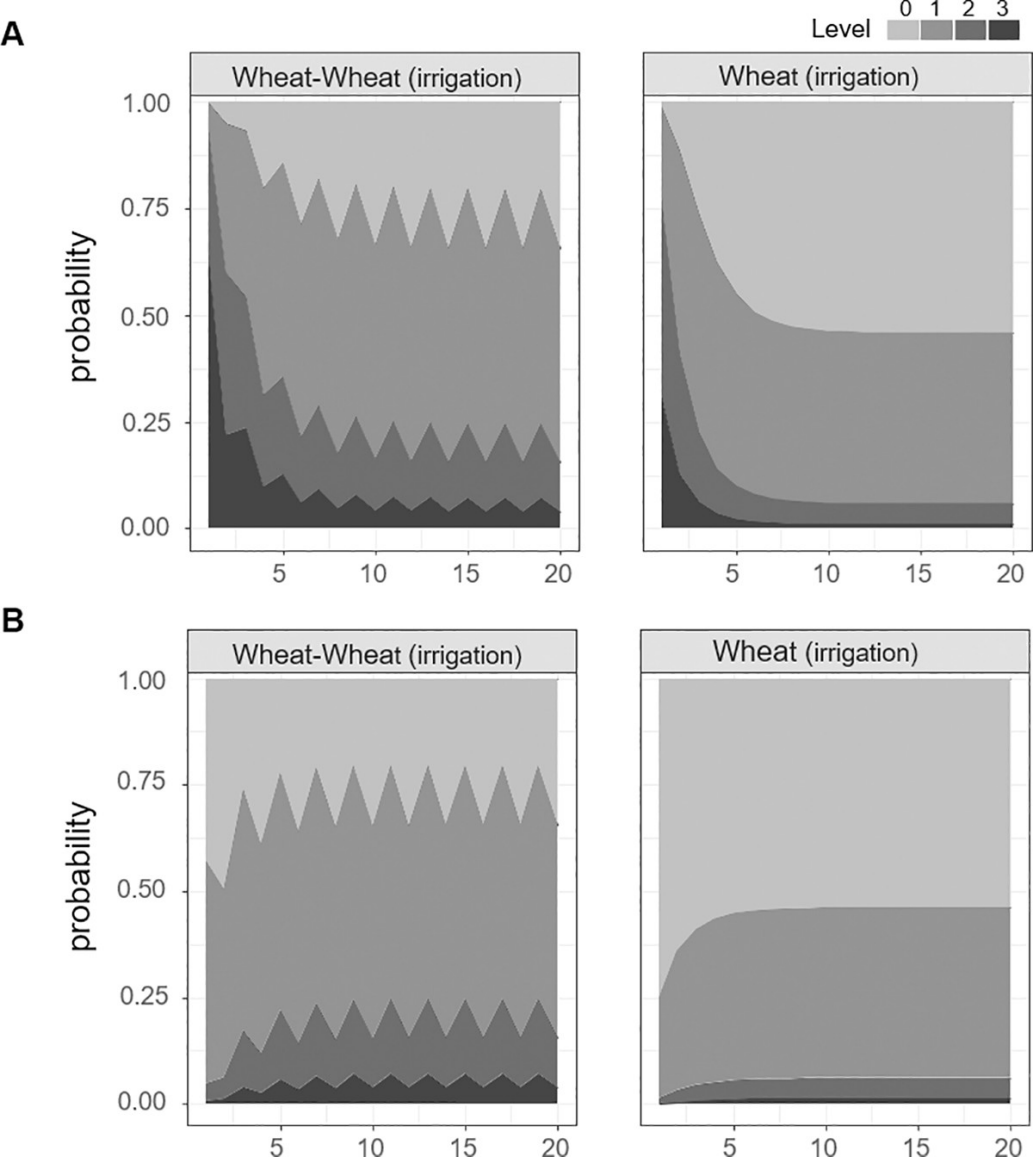
Projected state probabilities under repeated cycles of “wheat-wheat with summer irrigation” and “wheat with summer irrigation”. A) Initial coverage was set at level 0 (*A*. *fatua* is absent) and B) level 3 (high *A*. *fatua* coverage).

Coverage levels under all management options were projected to reach convergence in less than 10 years (Figs 3 and 4), with a notable change within the first 3 years. Under the “wheat” and “wheat-barley” strategies, the probability of *A*. *fatua* colonization (levels 1, 2, and 3) was greater than 0.9 (Fig 3 and Table 3). Meanwhile, farmland use also had a strong effect on *A*. *fatua* coverage; for example, the “paddy rice” cycle appeared to facilitate rapid convergence to level 0 in 1 year (Fig 3 and Table 3). Under the remaining cycle options, “wheat-other crop,” “wheat-paddy rice,” and “wheat-fallow with spring management,” the probabilities of level 0 or 1 coverage were almost 1 (Fig 3 and Table 3).

**Table 3:** Equilibrium probabilities of *Avena fatua* coverage under each rotation type.

Rotation	Farmland use	Lv0	Lv1	Lv2	Lv3
Wheat	Wheat	0.09	0.43	0.27	0.21
Wheat—Barley	Wheat	0.07	0.39	0.28	0.25
	Barley	0.06	0.38	0.29	0.27
Wheat—Other crop	Wheat	0.42	0.53	0.04	0.01
	Other crop	0.99	0.01	0.00	0.00
Wheat–Paddy rice	Wheat	0.43	0.53	0.04	0.01
	Paddy rice	1.00	0.00	0.00	0.00
Wheat—Fallow (management)	Wheat	0.41	0.53	0.05	0.01
	Fallow (management)	0.96	0.04	0.00	0.00
Wheat—Fallow	Wheat	0.22	0.55	0.17	0.06
	Fallow	0.38	0.49	0.10	0.03
Wheat (irrigation)	Wheat (irrigation)	0.54	0.40	0.05	0.01
Wheat—Wheat (irrigation)	Wheat	0.20	0.55	0.17	0.07
	Wheat (irrigation)	0.35	0.50	0.12	0.04

## Discussion

### Effects of each IWM strategy

The results of the transition matrix model obtained using accumulated coarse monitoring data demonstrated the contribution of each IWM strategy on *A*. *fatua* coverage and the effects of its repetition. The model projected that farmland use has a remarkable effect on coverage level of *A*. *fatua*, which tended to increase in “wheat” and “barley” fields, whereas fields planted with “paddy rice” and “other crops” drastically decreased the effect of coverage in the previous year. Barley is reported to be more competitive than wheat [25,43]; however, the results of this study suggest no differences in suppressing *A*. *fatua*. Selection of a cycle that incorporates other crops, paddy rice, or fallowing rather than wheat or barley is therefore recommended.

The “fallow” cycle option also had a strong effect on *A*. *fatua* coverage, although it increased the risk of increasing coverage compared with that of “paddy rice” and “other crops.” These findings suggest that the colonization risk of *A*. *fatua* is lower in cropped fields (other than those containing winter cereal) compared with fallow fields. Both the “fallow with spring management” and “fallow without spring management” options, which differed in terms of tillage, contributed to a decrease in *A*. *fatua* coverage; however, the effect was larger under “fallow with spring management.” This suggests that tillage during the booting to flowering stages is effective in breaking the life cycle of *A*. *fatua* and eliminating it. Mature weeds including *A*. *fatua* were sometimes able to survive spring tillage; thus, to prevent seed setting and dispersal, a management strategy that involves removing mature plants is important.

Fallowing could also help suppress *A*. *fatua*, although it risks increasing the invasion of other weed species. For example, in this study, weed species such as *Capsella bursa-pastoris* and *Equisetum arvense* were observed in place of *A*. *fatua*. *C*. *bursa-pastoris* is a common weed in vegetable fields in Japan [44], and *E*. *arvense* is known to cause damage to various types of agricultural fields [45]. Understanding the risk of invasion from other species and appropriate management is therefore important when considering fallowing as an IWM strategy.

### Effect of irrigation in wheat fields

It was previously reported that wet soil conditions contribute to the decay of buried seeds [27,28]. The results of this study suggest that “summer irrigation” could effectively suppress *A*. *fatua* abundance in wheat fields. In Honshu, rotation of paddy rice and wheat is a major cropping system, a decrease in *A*. *fatua* damage has been reported for fields using this system [30]. The results of our model were also in line with this.

Although summer irrigation of wheat fields is effective, paddy rice rotation rather than continuous wheat was able to more rapidly and more effectively eliminate *A*. *fatua* coverage. This is thought to reflect the effect of summer irrigation on the seed bank, whereas paddy rice reduces both the seed bank and breaks the life cycle of *A*. *fatua* via spring tillage and irrigation.

In this study, the effects of delayed sowing and temperature were not included in the model because they were found to be weaker than those of the remaining variables. Previous studies have suggested the effectiveness of removing *A*. *fatua* seedlings by delayed sowing [23], and the effects of warm temperatures on *A*. *fatua* emergence have also been pointed out [34]. In the present study, negative effects of delayed sowing and positive effects of temperature were detected. Thus, although both effects were relatively small compared with farmland use and summer irrigation, our results do not contradict the results of previous studies.

### Number of years required for *A*. *fatua* control

Crop rotation using other crops or fallowing is another effective method of weed control in wheat fields [20,21]. For example, planting barley instead of wheat is considered effective because it is more competitive [25], whereas winter fallow with tillage is thought to remove emerging *A*. *fatua* before seed maturation, helping to deplete the seed bank. Although the number of years required for crop rotation or fallowing have been unclear in spite of its importance, the results of our simulations provide information about it.

Simulations of repeated management revealed that farmland use in the form of rotation with “paddy rice” and “other crops” contributed to rapid *A*. *fatua* control in 1 year, even under the highest level of coverage (level 3). These findings suggest that changing farmland use only once can effectively eliminate *A*. *fatua* in fields where an outbreak has occurred. The remaining management options established a trend within 3 years and reached convergence in less than 10 years. These results suggest that our model can accurately estimate the effects of various management strategies and their cycles and can be used to aid decision-making on which strategies to select and how long to continue them.

## Conclusions

The results of this study suggest that the transition matrix model obtained using accumulated coarse monitoring data is a powerful tool for projecting the abundance and effects of multiple IWM strategies and their repetition on the suppression of *A*. *fatua*. Because few studies have used such simplified density data to consider weed dynamics, these findings contribute to weed management decision-making support by showing the usefulness of such simplified monitoring data. The results also highlight the potential risk of emergence and/or increased *A*. *fatua* coverage in crop fields, providing information on different management strategies and the recommended duration of each cycle option. Even on farms with a severe outbreak of *A*. *fatua*, just one significant change in farmland use, such as a change from wheat to vegetable or paddy rice could drastically reduce coverage and improve farm conditions. Thus, although management of *A*. *fatua* in crop fields requires IWM, our findings will help to support decision-making processes and facilitate efficient agricultural production.

## Supporting information

S1 Table The results of 425 visual observations. “site_id” corresponds to “Site ID” in Table 1.
(CSV)

## References

[1] Buhler DD. Challenges and Opportunities for Integrated Weed Management. Weed Sci Soc Am. 2002;50: 273–280.

[2] Harker KN, O’Donovan JT. Recent Weed Control, Weed Management, and Integrated Weed Management. Weed Technol. 2013;27: 1–11. 10.1614/wt-d-12-00109.1

[3] Neve P, Barney JN, Buckley Y, Cousens RD, Graham S, Jordan NR, et al Reviewing research priorities in weed ecology, evolution and management: a horizon scan. Weed Res. 2018;58: 250–258. 10.1111/wre.12304 30069065 PMC6055875

[4] Moss S. Integrated weed management (IWM): why are farmers reluctant to adopt non-chemical alternatives to herbicides? Pest Manag Sci. 2019;75: 1205–1211. 10.1002/ps.5267 30450751

[5] Liebman M, Baraibar B, Buckley Y, Childs D, Christensen S, Cousens R, et al Ecologically sustainable weed management: How do we get from proof-of-concept to adoption? Ecol Appl. 2016;26: 1352–1369. 10.1002/15-0995 27755749

[6] Chikowo R, Faloya V, Petit S, Munier-Jolain NM. Integrated Weed Management systems allow reduced reliance on herbicides and long-term weed control. Agric Ecosyst Environ. 2009;132: 237–242. 10.1016/j.agee.2009.04.009

[7] Florax RJGM Travisi CM, Nijkamp P. A meta-analysis of the willingness to pay for reductions in pesticide risk exposure. Eur Rev Agric Econ. 2005;32: 441–467. 10.1093/erae/jbi025

[8] Macé K, Morlon P, Munier-Jolain N, Quéré L. Time scales as a factor in decision-making by French farmers on weed management in annual crops. Agric Syst. 2007;93: 115–142. 10.1016/j.agsy.2006.04.007

[9] Blackshaw RE, Moyer JR, Harker KN, Clayton GW. Integration of Agronomic Practices and Herbicides for Sustainable Weed Management in a Zero-Till Barley Field Pea Rotation 1. Weed Technol. 2005;19: 190–196. 10.1614/wt-04-128r

[10] Anderson RL. A multi-tactic approach to manage weed population dynamics in crop rotations. Agron J. 2005;97: 1579–1583. 10.2134/agronj2005.0194

[11] Taylor CM, Hastings A. Finding optimal control strategies for invasive species: A density-structured model for Spartina alterniflora. J Appl Ecol. 2004;41: 1049–1057. 10.1111/j.0021-8901.2004.00979.x

[12] Freckleton RP, Hicks HL, Comont D, Crook L, Hull R, Neve P, et al Measuring the effectiveness of management interventions at regional scales by integrating ecological monitoring and modelling. Pest Manag Sci. 2018;74: 2287–2295. 10.1002/ps.4759 29024368 PMC6175144

[13] Jabran K, Mahmood K, Melander B, Bajwa AA, Kudsk P. Weed Dynamics and Management in Wheat 1st ed Advances in Agronomy. Elsevier Inc.; 2017 10.1016/bs.agron.2017.05.002

[14] CABI. Avena fatua, In: Invasive Species Compendium. Wallingford, UK: CAB International 2020 Available from: www.cabi.org/isc.

[15] Beckie HJ, Francis A, Hall LM. The Biology of Canadian Weeds. 27. Avena fatua L. (updated). Can J Plant Sci. 2012;92: 1329–1357. 10.4141/CJPS2012-005

[16] Heap I. Global perspective of herbicide-resistant weeds. Pest Management Science. 2014;70: 1306–1315. 10.1002/ps.3696 24302673

[17] Bajwa AA, Akhter MJ, Iqbal N, Peerzada AM, Hanif Z, Manalil S, et al Biology and management of Avena fatua and Avena ludoviciana: two noxious weed species of agro-ecosystems. Environ Sci Pollut Res. 2017;24: 19465–19479. 10.1007/s11356-017-9810-y 28766148

[18] O’Donovan JT, Harker KN, Turkington TK, Clayton GW. Combining cultural practices with herbicides reduces wild oat (Avena fatua) seed in the soil seed bank and improves barley yield. Weed Sci. 2013;61: 328–333. 10.1614/ws-d-12-00168.1

[19] Harker KN, O’Donovan JT, Turkington TK, Blackshaw RE, Lupwayi NZ, Smith EG, et al Diverse Rotations and Optimal Cultural Practices Control Wild Oat (Avena fatua). Weed Sci. 2016;64: 170–180. 10.1614/ws-d-15-00133.1

[20] Harker KN, O’Donovan JT, Irvine RB, Turkington TK, Clayton GW. Integrating Cropping Systems with Cultural Techniques Augments Wild Oat (Avena fatua) Management in Barley. Weed Sci. 2009;57: 326–337. 10.1614/ws-08-165.1

[21] Martin RJ, Felton WL. Effect of crop rotation, tillage practice, and herbicides on the population dynamics of wild oats in wheat. Aust J Exp Agric. 1993;33: 159–165. 10.1071/EA9930159

[22] Brown DA. Wild Oats: Progress in Cultural Control. Weeds. 1953;2: 295–299.

[23] Asai M, Yogo Y. Wheat seeding time, seeding rate and trifluralin application affect the control of wild oat (Avena fatua)(in Japanese with English title and abstract). J Weed Sci Technol. 2010;55: 158–166. 10.3719/weed.55.158

[24] O’Donovan JT, Harker KN, Clayton GW, Hall LM. Wild Oat (Avena fatua) Interference in Barley (Hordeum vulgare) is Influenced by Barley Variety and Seeding Rate. Weed Technol. 2000;14: 624–629. 10.1614/0890-037x(2000)014[0624:woafii]2.0.co;2

[25] Satorre EH, Snaydon RW. A comparison of root and shoot competition between spring cereals and Avena fatua L. Weed Res. 1992;32: 45–55. 10.1111/j.1365-3180.1992.tb01861.x

[26] Kirkland KJ. Weed Management in Spring Barley (Hordeum vulgare) in the Absence of Herbicides. J Sustain Agric. 1993;3: 95–104. 10.1300/J064v03n03_07

[27] Mickelson JA, Grey WE. Effect of soil water content on wild oat (Avena fatua) seed mortality and seedling emergence. Weed Sci. 2006;54: 255–262. 10.1614/ws-05-007r.1

[28] Kida Y, Asai M. Effect of summer flooding on wild oat and Italian ryegrass seed viability (in Japanese with English title). J Weed Sci Technol. 2006;51: 87–90.

[29] Burns EE, Lehnhoff EA, Mckenzie SC, Maxwell BD, Dyer WE, Menalled FD. You cannot fight fire with fire: a demographic model suggests alternative approaches to manage multiple herbicide-resistant Avena fatua. Weed Res. 2018;58: 357–368. 10.1111/wre.12315

[30] Asai M, Yogo Y. Occurrence and background of wild oats and Italian ryegrass in winter cereals in the Kanto- Tokai region (in Japanese with English title and abstract). J Weed Sci Technol. 2005;50: 73–81.

[31] Agricultural Production Bureau, Ministry of Agriculture, Forestry and Fisheries of Japan. Survey on the occurrence and damage of weeds in wheat and soybean production (in Japanese). 2013. Available from: https://www.maff.go.jp/j/seisan/ryutu/info/pdf/zassou-tyousa.pdf

[32] Asai M. Differential response to seed burial depth on seedling emergence of four Avena fatua L. populations in western Ibaraki, Japan (in Japanese with English title and abstract). J Weed Sci Technol. 2020;65: 103–109.

[33] Page ER, Gallagher RS, Kemanian AR, Zhang H, Fuerst EP. Modeling site-specific wild oat (Avena fatua) emergence across a variable landscape. Weed Sci. 2006;54: 838–846. 10.1614/ws-05-142r1.1

[34] Blanco AM, Chantre GR, Lodovichi M V, Bandoni JA, López RL, Vigna MR, et al Modeling seed dormancy release and germination for predicting Avena fatua L. field emergence: A genetic algorithm approach. Ecol Modell. 2014;272: 293–300. 10.1016/j.ecolmodel.2013.10.013

[35] Ohno H, Sasaki K, Ohara G, Nakazono K. Development of grid square air temperature and precipitation data compiled from observed, forecasted, and climatic normal data (in Japanese with English title). Clim Biosph. 2016;16: 71–79.

[36] Kominami Y, Sasaki K, Ohno H. User’s manual for The Agro-Meteorological Grid Square Data, NARO Ver.4 (in Japanese). NARO. 2019; 67.

[37] Freckleton RP, Sutherland WJ, Watkinson AR, Queenborough SA. Density-structured models for plant population dynamics. Am Nat. 2011;177: 1–17. 10.1086/657621 21126176

[38] Agresti A. Categorical Data Analysis, Second Edition John Wiley & Sons, Inc; 2002 10.1002/0471249688

[39] Christensen RHB. ordinal—Regression Models for Ordinal. In: Data. R package version 2019.12–10. 2019. Available from: https://cran.r-project.org/package=ordinal.

[40] R Core Team. R: A language and environment for statistical computing. R Foundation for Statistical Computing Vienna, Austria; 2018 Available from: http://www.r-project.org/.

[41] Akaike H. A new look at the statistical model identification. IEEE Trans Automat Contr. 1974;19: 716–723.

[42] Mertens SK, Van Den Bosch F V., Heesterbeek JAP. Weed populations and crop rotations: Exploring dynamics of a structured periodic system. Ecol Appl. 2002;12: 1125–1141. 10.1890/1051-0761(2002)012[1125:WPACRE]2.0.CO;2

[43] Blackshaw R, O’Donovan JT, Harker KN, Li X. Beyond herbicides: new approaches to managing weeds. Proc ICESA Conf. 2002; 305–312. ISBN 0-9733880-1-3.

[44] Shibayama H, Ogawa A. Effects of Environmental Factors on the Emergence of Capsella bursa-pastoris (L.) Medik. in Upland Crop Fields in Northern Saga Prefecture. J Weed Sci Technol. 2000;45: 207–213.

[45] Nakatani K. Basic studies on reproductive characteristics and environmental responses in Equisetum arvense (in Japanese with English title). J Weed Sci Technol. 2015;60: 158–165. 10.3719/weed.60.158

